# Imaging Markers for Normal Pressure Hydrocephalus: An Overview

**DOI:** 10.3390/biomedicines11051265

**Published:** 2023-04-24

**Authors:** Efstratios-Stylianos Pyrgelis, Georgios Velonakis, Sokratis G. Papageorgiou, Leonidas Stefanis, Elisabeth Kapaki, Vasilios C. Constantinides

**Affiliations:** 1 1st Department of Neurology, School of Medicine, National and Kapodistrian University of Athens, Eginition Hospital, Vass. Sophias Ave. 74, 11528 Athens, Greece; 2 Neurochemistry and Biological Markers Unit, 1st Department of Neurology, School of Medicine, National and Kapodistrian University of Athens, Eginition Hospital, Vass. Sophias Ave. 74, 11528 Athens, Greece; 3 Research Unit of Radiology, 2nd Department of Radiology, Medical School, National and Kapodistrian University of Athens, “Attikon” University General Hospital, Rimini 1, Chaidari, 12462 Athens, Greece

**Keywords:** idiopathic normal pressure hydrocephalus, imaging, markers

## Abstract

Idiopathic bormal pressure hydrocephalus (iNPH) is a neurological syndrome that clinically presents with Hakim’s triad, namely cognitive impairment, gait disturbances, and urinary incontinence. The fact that iNPH is potentially reversible makes its accurate and early diagnosis of paramount importance. Its main imaging characteristic is the dilation of the brain’s ventricular system and the imaging parameters are also included in its diagnostic criteria along with clinical data. There is a variety of different modalities used and a great number of imaging markers that have been described while assessing iNPH patients. The present literature review attempts to describe the most important of these imaging markers and to shed some light on their role in diagnosis, differential diagnosis, and possibly prognosis of this potentially reversible neurological syndrome.

## 1. Introduction

Hydrocephalus is defined as the abnormal enlargement of the brain’s ventricles, which could be the result of increased production, decreased absorption, or obstruction of the normal flow of cerebrospinal fluid (CSF). There are two main types of hydrocephalus: obstructive and communicating. Normal pressure hydrocephalus is a subtype of communicating hydrocephalus that occurs more frequently in the elderly. It is distinguished into secondary, which can be the result of a specific cause (e.g., subarachnoid hemorrhage, brain injury, infection, tumor, etc.) and idiopathic normal pressure hydrocephalus (iNPH), which has no identifiable cause [1,2,3].

The incidence of iNPH ranges in studies from 1.8 to 7.3/100,000 and its prevalence ranges from 0.41% to 3.5% in individuals older than 65 years [4], whereas its frequency tends to increase with age [4,5]. This syndrome is clinically characterized by Hakim’s triad of symptoms: gait disorders, cognitive impairment, and urinary incontinence [6,7]. There are various theories about the pathogenesis of iNPH, most of which include the disruption of circulation of CSF, which leads to a deceleration of its absorption and enlargement of the ventricular system as the common pathway [8,9]. This syndrome Is often difficult to differentiate from vascular dementia, neurodegenerative dementias, and parkinsonian syndromes, whereas sometimes these pathologies are concomitant [9,10,11,12].

The most well-established diagnostic test and at the same time a potential prognostic tool of successful response to treatment with shunt surgery is the so-called tap test, which involves the removal of CSF and the evaluation of possible subsequent clinical improvement [9,13].

According to the latest updates of guidelines for the management of iNPH endorsed by International and Japanese experts on the field, the diagnosis of iNPH requires both concrete clinical and imaging criteria [14,15]. Thus, brain imaging in patients with iNPH is essential to diagnosis.

Therefore, we conducted the present narrative review, based on research and conceptual studies, in order to present available literature data regarding neuroimaging markers in patients with iNPH and outline the general principles regarding their role in diagnosis, differential diagnosis, and perhaps prognosis of this potentially reversible neurological disorder.

## 2. Materials and Methods

A search was performed to select studies in PUBMED database from study inception to September 2022. There were no restrictions performed during the literature search. Keywords used to query the database included: “idiopathic normal pressure hydrocephalus”, “imaging”, and “markers”. Relevant information was also extracted from the appropriate references of all included articles and from other types of published manuscripts such as reviews and case studies. Finally, the findings were critically summarized and presented in the present review.

## 3. Results

### 3.1. Imaging Markers Used in the Diagnosis of iNPH

In the literature, there is a pleiad of imaging signs and markers used to support the clinical suspicion of iNPH that can be evaluated using either computed tomography (CT) or magnetic resonance imaging (MRI). Some of the most commonly used include dilated ventricles, narrow sulci at the high convexities, and signs of hyperdynamic CSF flow [16].

The most established index for assessment of ventricular dilation is the Evans index (EI). It was first described by Evans in 1942 and is defined as the ratio of the frontal horn diameter (FHD) (the maximum distance between the lateral margins of the frontal ventricles) to the inner skull diameter (ISD) (the maximum lateral-to-lateral distance between the inner laminae of the parietal bones on the same transverse slice) [17,18]. The diagnostic criteria of iNPH require an EI of 0.3 or greater [14]. Missori et al., in 2016 concluded that, although normal aging can enlarge the ventricles, EI values greater than 0.30 should not be attributed to normal aging as they most likely reflect an underlying neurological pathology [19]. Despite the above, another study by Brix et al., (2017) showed that there are great discrepancies of EI measurements among healthy old people, and, thus, a cut-off value of 0.3 may be difficult to discriminate between normal and enlarged ventricles, proposing higher cut-off values (0.32–0.37), which vary depending on gender and age [20].

Callosal angle (CA) is a marker measured on a coronal image perpendicular to the anterior commissure–posterior commissure (AC-PC) plane at the level of the posterior commissure, which has been found to have lower values in iNPH than in ex vacuo ventriculomegaly, possibly because of the elevation of the dilated lateral ventricles [14,21]. The simplified CA is another imaging marker suggested by Cagnin et al., (2015). It is measured on the coronal sections that pass through the corpus callosum midpoint. The vertex of the angle is placed in the inferior point of the corpus callosum and the sides tangential to the lateral ventricles [22]. The anterior callosal angle (ACA) is a more anterior variant of this marker, measured at the level of the AC, and has also been proposed as an iNPH imaging marker [23]. 

Additionally, dilatation of the temporal horns of the lateral ventricles is also an early imaging marker of hydrocephalus [24], while Kojoukhova et al., (2015) found that narrower temporal horns are associated with a higher probability of iNPH diagnosis [25].

Another proposed imaging marker, assessing ventricular size, is the anteroposterior diameter of the lateral ventricle index (ALVI), calculated as the ratio of the lateral ventricle diameter measurement to the maximal width of the anteroposterior inner diameter of the skull in the same slice. The ALVI seems to better reflect the ventricular volume than the EI [26].

The widest diameter of the third ventricle, between the anterior and posterior commissures, on coronal slice, as well as the widest anteroposterior midline diameter of the fourth ventricle determined along a line perpendicular to the posterior border of the brain stem on sagittal slices, focally enlarged sulci on coronal slices, dilation of Sylvian fissures on coronal slices and high-tight-convexity sulci on trans-axial slices, have also been used in evaluation of iNPH [10,27]. The latter has been studied in detail by Sakaki et al., (2008), who reported that the presence of narrow sulci at the high convexity or midline can be detected with sufficient accuracy both in axial and coronal slices in brain MRI and is useful in the diagnosis of iNPH [28].

Other indexes that have been proposed for the neuroimaging assessment of iNPH include the z-EI, which is defined as the ratio of FHD measured at the level of the foramen of Monro to the maximum supratentorial intracranial diameter (MSID) (measured bone-to-bone on the midsagittal section) [29]. The brain-to-ventricle ratio (BVR) has been defined as the proportion of the maximum vertical width of the supraventricular brain (SVW) to the cella media of the lateral ventricle width (CMW) [30] and the cella media-to-temporal horn ratio (CTR) that is measured as the ratio between the CMW and temporal horns width (THW) [31]. Callosal-ventricular distance (CVD) has been defined as the vertical distance from the inferior point of the corpus callosum to the line connecting the roofs of the lateral ventricles on coronal sections; callosal-commissural distance (CCD) has been defined as the distance between posterior commissure and undersurface of the corpus callosum on a line parallel to posterior brain stem margin on the mid-sagittal section; and callosal height (CH) has been defined as the maximum perpendicular distance between the bi-callosal line and undersurface of the corpus callosum on the mid-sagittal section [32]. Frontal and occipital horn ratio (FOR) has been defined as the average of the maximum frontal and occipital horn width divided by the interparietal distance and modified cella media index (mCMI) has been defined as the ratio of the maximal width of both cella media and the maximum inner diameter of the skull at the same axial slice. In a study using the above two indexes, along with other linear measurements, and comparing them to volumetric study of the ventricles, it has been shown that mCMI is more accurate than EI, FOR, and other simple linear measurements [33].

Periventricular hyperintensities in MRIs, which have also been used as an iNPH imaging marker, are T2-hyperintense white matter lesions next to the frontal and occipital horns of the lateral ventricles, which are relatively usual in old patients [34]. 

Holodny et al., (1998) reported that focal dilation of the fissures and sulci could represent reservoirs of CSF similar to the ventricular system [24]. Disproportionately enlarged subarachnoid space hydrocephalus (DESH) refers to a visual assessment of the enlarged subarachnoid spaces at the Sylvian fissures disproportionately to the subarachnoid spaces at the superior parasagittal convexity [10,35]. Kojoukhova et al., (2015) also showed that disproportion between supra-sylvian and sylvian subarachnoid spaces is the most useful imaging marker for iNPH diagnosis [25].

The flow void phenomenon (FVP) in the aqueduct and fourth ventricle, also known as flow void sign, has also been proposed in iNPH imaging evaluation. It is graded using a scale initially proposed by Algin et al., (2009) and later modified by Virhammar et al., (2014) [16,36]. Intense FVP, although characteristic for iNPH, is not pathognomonic for it [36]. Focal bulging of the roof of the lateral ventricles and focal narrowing of the aqueduct have also been used in iNPH diagnostic evaluation [16]. Virchow–Robin spaces (VRSs) are known to increase with age and is often associated with vascular dementia (VD), although it seems to have high incidence in patients with iNPH and does not seem to be able to discriminate iNPH patients from age-matched controls [37].

Diffusion Tensor Imaging (DTI) can be used to characterize the magnitude, the degree of anisotropy, and the orientation of directional diffusion. It is a technique with great potential in typifying microstructural alterations caused by neuropathology. The most common measures of DTI are mean diffusivity (MD), fractional anisotropy (FA), radial diffusivity (D r), and axial diffusivity (D a) [38]. DTI has been used to assess alterations in white matter tracts and its implementation on iNPH revealed increased FA and decreased MD in the corticospinal tract [39,40]. Reduced FA in the internal capsule and the corpus callosum has also been revealed [41]. FA and axial diffusivity of the ventrolateral (VL) and ventroposterolateral (VPL) nuclei of thalamus were increased among iNPH patients compared to healthy individuals [42]. Quantitative diffusion microstructure imaging (DMI) infers more specific tissue properties than conventional DTI. Elevated water content has been found, using DMI, in periventricular hyperintensities. Given the fact that this could be the imaging correlate of pathologic fluid accumulation, it might be used as an imaging biomarker of iNPH in the future [43,44]. Volumetric variables using MRI have also been analyzed in iNPH patients and they seem to be particularly useful in differential diagnosis and prognosis of these patients [45,46].

A study using phase-contrast MRI has shown that aqueductal CSF flow is strongly correlated with the total ventricular volume and the third ventricle width and that aqueductal stroke volume (ASV) is linearly correlated with aqueductal lumen area. However, aqueductal CSF flow did not seem to correlate with the tested hydrodynamic parameters [47]. A study using phase-contrast MRI on animal models of adult chronic communicating hydrocephalus has drawn the following conclusions. During the first 60 days of the study, a rapid ventricular enlargement, which positively correlated with the ASV increase, was observed. However, after this period less ventricular enlargement and heterogeneous measurements of the ASV were noticed [48].

While commonly used radiologic markers are of value, they can be aggregated into a score for better sensitivity and specificity. The DESH score is a composite imaging index consisting of five markers, namely ventriculomegaly, dilated sylvian fissures, high tight convexity, acute callosal angle, and focal sulcal dilation. Each of these 5 markers are rated from 0 to 2 [49]. Another structured scale for the overall evaluation of iNPH imaging data has been proposed by Kockum et al.,—the so-called Radscale [50,51]. It is a radiological scale that involves seven imaging features of iNPH, namely EI, CA, focally enlarged sulci, narrow sulci, dilation of temporal horns, dilation of Sylvian fissures, and periventricular pathologic intensity. The aforementioned features are graded appropriately as 0, 1, or 2, apart from focally enlarged sulci and dilation of the Sylvian fissures, which are graded as 0 or 1 [51].

Ringstad et al., (2017) studied the glymphatic function, which is the transport and clearance of substances in the central nervous system through channels consisting of glial cells, in patients with iNPH, administering a paramagnetic agent and using MRI to evaluate its clearance in several time-points. They found that glymphatic clearance in iNPH patients was reduced compared to the controls. Despite the above, they also concluded that the same could happen in other dementias such as Alzheimer’s disease (AD) making the usefulness of the aforementioned finding doubtful [52].

Magnetic resonance elastography (MRE) has been proposed as a helpful tool in differentiating dementia from normal aging. In MRE, iNPH is found to have decreased brain stiffness in the periventricular areas and increased stiffness in parietal and occipital areas; however, the results for the whole brain were inconclusive [53]. 

Cerebral blood flow (CBF) and regional cerebral blood flow (rCBF) using single photon emission computed tomography (SPECT) have been found, according to some studies, to be impaired in iNPH patients [12,54,55]. A new technique named arterial spin labeling perfusion, is able to mark and track the blood perfusing the brain without using a paramagnetic agent. Using this technique, a study by Virhammar et al., (2017) showed decreased blood supply in the periventricular white matter, basal ganglia, and thalamus in iNPH patients [56].

Dopamine transporter single-photon emission (DaT-Scan), despite being a modality that mainly aims to detect dopaminergic deficit in patients with Parkinson’s disease (PD) and atypical parkinsonian syndromes, has also been found to be impaired in iNPH patients by a few studies [57,58].

Finally, new techniques that make use of artificial intelligence (AI) have been proposed. Quon et al., created a deep learning model for automatic ventricle volume calculation, based on T2-weighted MRI images, that is quick and accurate enough. The main disadvantage of this method is that the model is based on the data of pediatric patients, making the implementation on old patients with iNPH relatively uncertain [59]. Other studies using AI involve using machine learning to select DESH signs and measure ventricular volumes, or correlating decreased volumes of the caudate, thalamus, putamen, pallidum, and hippocampus to cognitive impairment in iNPH [60,61].

### 3.2. Imaging Markers Used in iNPH Differential Diagnosis

As iNPH shares clinical findings with VD, a lot of neurodegenerative dementias and parkinsonian syndromes such as AD, dementia with Lewy bodies (DLB), frontotemporal dementia (FTD), PD, multiple system atrophy parkinsonian type (MSA-P), progressive supranuclear palsy (PSP), and corticobasal degeneration (CBD), a differential diagnosis among these disorders is often challenging [62,63]. Thus, markers that have some disease-specificity would be of great value [63].

CA is a marker that is helpful in distinguishing patients with iNPH (lower values), from those with ex vacuo ventriculomegaly [14]. A CA < 90° is a rough cut-off value for differentiating the dilation of ventricular system observed in iNPH from the pattern of brain atrophy observed in Alzheimer’s disease [21]. Simplified CA in brain MRI, proposed by Cagnin et al., (2015), is a useful marker, and is able to assist in differentiating among iNPH, AD, and DLB [22]. ACA has shown high accuracy in discriminating iNPH patients from AD patients and healthy individuals [23]. z-EI followed by CMW, FHD, BVR, SVW, CA, and DESH are better at discriminating between iNPH patients and healthy individuals [32].

Fällmar et al., (2021) have found that several imaging markers including callosal angle, enlarged Sylvian fissures, and focally enlarged sulci, maintain diagnostic accuracy when comparing iNPH to its mimics such as VD. Despite the fact that callosal angle was found to be the single imaging marker with the highest diagnostic accuracy in differentiating iNPH from its mimics, the authors suggested that a simplified composite imaging scale consisting of EI, CA, focally enlarged sulci, and enlarged sylvian fissures could be useful towards this direction [62].

According to some studies, in iNPH, atrophy of the hippocampus is milder and the dilation of the parahippocampal sulcus is less significant than in AD [64,65]. Moreover, patients with iNPH have been found to have dilated ventricles and relatively small perihippocampal fissures, while AD patients had dilated perihippocampal fissures and ventricles significantly smaller than those in iNPH patients [64]. However, according to a more recent study by Sakurai et al., (2021), hippocampal atrophy along with temporal horn enlargement seem to be typical radiological findings of severely demented patients, with either disproportionately enlarged subarachnoid-space hydrocephalus or later stages of AD as underlying pathology and are therefore not appropriate markers for differentiating between these diseases [66].

The periventricular white matter lesions (WML), volume (PWML-V), and deep WML volume (DWML-V) can be used to differentiate iNPH from AD. The DWML-V has been found to be dominant in iNPH, while the PWML-V is found in AD and healthy individuals. These differences could reflect diminished CSF and Aβ clearance in iNPH [67]. On the other hand, periventricular hyperintensity (PVH) and deep white matter hyperintensity (DWMH) in brain MRIs were not able to differentiate between iNPH and Binswanger disease (BD) patients [68].

Kim et al., (2011) studied MRI DTI in iNPH patients before operation and found that in these patients FA was significantly higher in the posterior limb of the internal capsule than patients with AD and VD and healthy subjects. They also found that MD in the anterior periventricular white matter, the anterior limb of the internal capsule, and the superior longitudinal fasciculus was significantly higher in iNPH patients before operation in comparison with AD patients and healthy individuals, but lower when compared to VD patients [69]. Ivkovic et al., (2013), based on MRI DTI, found that using a MD histogram is helpful in differentiating among NPH, AD, and PD [70]. Another recent study used DTI on the area of corpus callosum, which is considered to be involved in iNPH, AD, and PSP. Corpus callosum integrity in iNPH was evaluated based on the spatial distribution of DTI-derived principal diffusion direction orientation (V1) and the following results were found by the authors: V1 distribution in corpus callosum splenium is different in iNPH compared to AD and PSP, while MD and FA had similar alterations in all aforementioned neurologic disorders and V1 is a more accurate marker than splenial volume for the differential diagnosis of these disorders [71]. It has also been found that FA in the anterior thalamic radiation and the minor forceps was lower in iNPH patients than in the PD patients [72]. Another study revealed that iNPH patients presented higher MD than AD patients and healthy individuals in the following areas: corpus callosum and middle cerebellar peduncle; external capsule, anterior corona radiate and superior longitudinal fasciculus blaterally; and left thalamic radiation [73]. These findings could mean that microstructural changes in white matter, evaluated using DTI, could be an imaging marker for differential diagnosis between AD and iNPH [73]. According to another study, it was found that increased MD of the superior thalamic radiation along with ventricular volume measurement resulted in good discrimination among iNPH, AD, and age-matched healthy individuals and that the ratio of ventricular to sulcal CSF is significantly greater in iNPH than in AD and age-matched healthy individuals [74]. Quantification of water content and intra-/extra-axonal volume fractions of periventricular hyperintensities using DMI could also prove to be able to assist in the differential diagnosis of T2 hyperintensities among iNPH and other neurological disorders [44].

Midbrain morphology in iNPH may resemble that of PSP. Established MRI markers of midbrain and superior cerebellar peduncle (SCP) atrophy cannot confidently differentiate PSP from iNPH [75]. The midbrain atrophy in PSP as well as the enlargement of the third ventricle in iNPH both lead to magnification of the interpeduncular cistern and subsequently smaller midbrain area, which can be measured on MRI midsagittal plane [76]. Hummingbird sign, an imaging sign of PSP reflecting midbrain atrophy with sparing of pons, could also been found in a great percentage of iNPH patients. Thus, when this sign is present, the other iNPH imaging signs should be evaluated before PSP suspicion is risen [77]. According to another study, the combination of EI and CA markers helped to discriminate PSP patients with marked ventricular dilatation from patients with iNPH, which could benefit from shunt surgery [78]. Magnetic Resonance Hydrocephalic Index (MRHI) is a linear imaging marker that is calculated as the ratio of the collateral trigones width (CTW) to the inner skull diameter measured on the same axial slice. This marker, along with automated ventricular volumetry (AVV), has shown high accuracy in differentiating iNPH from PSP [46]. Furthermore, Ugga et al., (2020) investigated the potential role of the interpeduncular angle (IPA), calculated as the angle formed by the posterior half of the cerebral peduncles at the level of the mammillary bodies or immediately below, which had significantly higher values in iNPH than in PSP patients, despite some overlap between the two groups [76].

Table 1 summarizes the imaging markers that are useful in the differential diagnosis of iNPH from its mimics.

### 3.3. Imaging Markers of iNPH with Possible Prognostic Value

It has been found that both EI higher than 0.3 and more acute CA (<90°) might correlate with better surgical outcome of iNPH patients [16,21], while more acute CA as a single marker has also been associated to tap test and shunt responsiveness [16,79,80]. Another study investigated the difference in EI and CA before and after surgical treatment with lumbo-peritoneal (LP) shunt placement. A significant decrease in CA and EI measurements in the early postoperative period could be an indicator of the possibility of iNPH patients to benefit from a surgical treatment and be clinically improved. Interestingly, postoperative imaging assessment predicted improvement of the gait disturbances and urinary incontinence but was unable to predict improvement of cognitive symptoms [81]. Virhammar et al., (2018) found that CA increased, and ventricular volume decreased after shunt surgery, with the alteration of CA being most explicit, indicating that this imaging marker could be used as an indirect tool to evaluate shunt function [82]. According to another study, neither CA nor cingulate sulcus sign were able to predict shunt responsiveness [83]. ACA has been significantly correlated with the improvement in gait and balance following shunt placement, suggesting that it could be used, along with the CA, as a possible predictor of shunt responsiveness in iNPH [84].

Preoperational high tight convexity, callosal angle, and Sylvian fissure dilation have been significantly associated with the clinical improvement one year following surgery when simple linear regression was implemented, while with multiple linear regression only high tight convexity seemed to predict that improvement [85]. Furthermore, wide temporal horns have been associated with positive outcome after shunt placement [16]. A more recent study has shown that high tight convexity, dilated Sylvian fissures and focally dilated sulci have also been correlated significantly with subjective shunt response at 1-month follow-up [86].

The load of white matter lesions has been negatively correlated to the tap test response [87]. Improvement of the load of white matter lesions after surgical treatment has failed to lead to firm conclusions regarding shunt responsiveness [88,89]. Nevertheless, according to another study, plentiful PVH and DWMH preoperatively as well as reduction in PVH postoperatively correlated with improvement in gait and cognitive performance after shunt placement [68], whereas a more recent study has shown that a post-shunt decrease of periventricular white matter hyperintensities may be a potential marker for shunt responsiveness [90]. 

The presence of “empty sella” sign and “mismatch” sign (disproportionally narrowed CSF space at the high convexity and dilatated Sylvian fissures) were correlated to positive tap test responder status [91]. Virhammar et al., (2014) positively correlated occurrence of disproportionately enlarged subarachnoid space hydrocephalus to shunt responsiveness [16], whereas Craven et al., (2016) found that although DESH sign had a relatively high positive prognostic value, it should not be used to exclude patients from shunt surgery due to its low negative prognostic value [92]. Moreover, Hong et al., (2018) found that good response to treatment with shunt placement was associated with fewer lacunes, and higher incidence of disproportionately enlarged subarachnoid spaces on preoperative MRI compared to the patients that did not respond [93]. 

A study by Ng et al., (2009), assessing MRI biomarkers in iNPH, showed a strong correlation between shunt response and decrease in apparent diffusion coefficient (ADC), supporting the hypothesis that water accumulation in the cerebrum is the major cause for the symptoms of iNPH. Additionally. a frequent presence of subdural hemorrhage among patients that did not respond well to shunt surgery raises suspicion of decreased compliance as the other major cause [94]. Agerskov et al., (2020), studying diffusion and perfusion MRI images, found a postoperative increase in ADC and highlighted a correlation between postoperative increase in rCBF in midbrain and pons and clinical improvement [95].

It has been suggested that changes in the quantitative value of FA, using DTI technique on MRI, after shunt placement have been associated with a degree of clinical improvement [96]. According to Tsai et al., (2018), the combination of FA of the VPL nucleus with CSF peak systolic velocity has proven to be very accurate in predicting tap test responsiveness and could thus be used as a potential prognostic biomarker [42]. Kang et al., (2016) found that tap test non-responders had lower FA in the left anterior thalamic radiation (ATR), left cingulum-hippocampus (CgH) and left inferior fronto-occipital fasciculus (IFO) and higher D a, D r, and MD in the left CgH and left inferior longitudinal fasciculus (ILF) than tap test responders. FA values in the ATR (bilateral), corticospinal tract (right), IFO (bilateral), and ILF (bilateral) were negatively correlated with Unified Parkinson’s Disease Rating Scale motor scores. In the right CgH, FA values showed significant correlations with cognitive scores; and specifically, a positive one with the Korean-Mini Mental State Examination scores and a negative one with Clinical Dementia Rating Scale scores. These findings might suggest that microstructural changes of white matter, as evaluated using DTI, could be useful in the prediction of tap-test responder status and that specific patterns of such changes may reflect specific symptom domains in iNPH patients [97]. Decreased FA in the splenium of the corpus callosum and right external capsule has been, according to another study, associated with gait dysfunction [73]. Thus, DTI may assist in understanding the pathophysiology of gait disturbances in iNPH [73]. Another study that implemented DMI techniques on iNPH patients showed that the orientational coherence within the corticospinal tracts was higher in patients than in healthy individuals preoperatively, while the normalization trend that presented after shunt surgery could reflect recovery. The estimated axon density was lower in iNPH patients than in healthy individuals and was not altered after surgery, proposing it as a possible biomarker of permanent neuronal damage [43]. FA in the anterior thalamic radiation has been significantly associated with gait disturbances in patients with iNPH and PD [72].

Phase-contrast MRI can quantify aqueductal CSF flow. Furthermore, the combination of this measurement with relative imaging parameters such as CSF flow void may be helpful in predicting shunt responsiveness [29,98,99]. Despite the above, a standalone ASV has not proven to be an adequate predictor of shunt responsiveness [47]

Luikku et al., (2016) and Todisco et al., (2020) concluded that none of the preoperative imaging markers could accurately predict the outcome following shunt surgery [90,100], whereas Agerskov et al., (2019) concluded that none of the MRI markers should be used as exclusion criteria from shunt surgery [27].

Regarding DESH score, it was positively correlated to shunt improvement possibility, with iNPH patients who were not improved regarding the modified Rankin Scale (mRS) score having a lower DESH score [49]. DESH score has also been correlated significantly with subjective shunt response at 1-month follow-up [86]. Nevertheless. according to other studies, DESH score could not predict shunt responsiveness [83,101].

Radscale implemented on iNPH patients’ brain CT or MRI, according to a retrospective study by Carlsen et al., 2021, has shown moderate discrimination for shunt responder status but it is unable to be the only tool used for selecting good candidates for shunt placement [102]. Another recent study showed that Radscale total-score was significantly higher in patients that responded well to shunt surgery [103]; whereas Chen et al., found no significant differences in Radscale total score between shunt surgery responders and non-responders [101]. Laticevschi et al., (2021) found that Radscale total score was similar between tap test responders and non-responders [104]. This finding was confirmed by another recent study that implemented Radscale on iNPH patients’ brain MRIs [80]. 

In a study comparing volumetric 3-dimentional (3D) images of brain MRI among iNPH tap test responders, iNPH tap test non-responders and AD patients, tap test non-responders were found to have statistically significant cortical thinning in the left superior frontal gyrus and widespread cortical thinning in most brain areas, compared to responders. AD patients showed statistically significant cortical thinning in superior and medial frontal gyrus, left precentral gyrus, postcentral gyrus, paracentral lobule, precuneus, and superior parietal lobule compared to tap test responders. Patterns of cortical thinning between AD patients and tap-test non-responders did not differ significantly [45]. Differences in cortical thickness correlated with tap test responder status for patients with iNPH may suggest a pattern of cortical thinning in patients with enlarged ventricles as possible imaging marker for the prediction of tap test responsiveness. [45].

In a study comparing the prognosis between iNPH patients with concomitant AD (certified by CSF biomarkers) and pure iNPH patients, the following conclusions were drawn. At baseline, there were no significant differences in the rCBF between the groups in most regions except for the putamen. In post-shunt evaluation of iNPH patients with concomitant AD, there was no significant improvement in rCBF in any brain region, while in pure iNPH patients there was a significant increase in rCBF in the putamen, amygdala, hippocampus, and para-hippocampal gyrus [105].

DWMH and PVH have been hypothesized to be related to CSF sulfatide and demyelination, and CSF neurofilament protein (NFL) and neuronal axonal dysfunction accordingly [68]. Postoperatively, a greater decrease in NFL correlated to a larger reduction in PVH and a more significant clinical improvement [106]. Using MR spectroscopy postoperatively, increased total choline and decreased myo-inositol in the frontal DWM was found, which could be correlated with clinical improvement [107].

Regarding functional neuroimaging, post-surgical improvement in 18F FDG PET glucose cortical metabolism may be considered a useful imaging marker for the assessment of shunt responsiveness [108]. Postsynaptic D2 receptor hypoactivity in the dorsal putamen has been associated with the severity of kinetic symptoms in iNPH [109]. Meanwhile, a study applying DaT-Scan pre- and post-operatively in iNPH patients revealed that the dopaminergic deficit normalized after shunt placement, proposing DaT-Scan as a potentially useful marker of iNPH pathology progression [110].

Table 2 summarizes the potential prognostic value of various preoperative iNPH imaging markers.

## 4. Discussion

In this narrative review, we studied the available data from the English-speaking literature on the use of imaging markers in diagnosis, differential diagnosis, and possibly prognosis of iNPH patients.

As mentioned above, the majority of the imaging markers applied during the evaluation of iNPH patients can be implemented either on CT or MRI. MRI has several advantages, however. It can better reveal periventricular white matter changes, as it has superior contrast regarding soft tissue and it can also include more sophisticated techniques such as phase-contrast MRI CSF flow study that can demonstrate CSF pulsatile flow during the cardiac cycle, by which stroke volume and flow velocity can be calculated, and DTI can be used to characterize the magnitude, the degree of anisotropy, and the orientation of directional diffusion of brain regions of interest [38,111,112].

Due to significant clinical and/or radiological overlap of iNPH with several other neurological disorders, markers with specificity for iNPH that are able to assist in differential diagnosis among these diseases are of paramount importance. CA and its variants such as simplified CA or ACA seem to be helpful in differentiating iNPH from ex vacuo ventriculomegaly, AD, VD, DLB, and PSP [14,22,23,62,78]. Other markers such as enlarged Sylvian fissures and focally enlarged sulci as well as a simplified composite imaging scale consisting of EI, CA, focally enlarged sulci, and enlarged sylvian fissures have also been considered useful in differential diagnosis of iNPH from its mimics [62]. The extent and pattern of hippocampal atrophy along with peri-hippocampal fissures dilation can assist in discriminating iNPH from AD [24,64,65]. The pattern of WML can be used for differentiation from AD—but not from BD [67,68]. DTI measures are also useful in discrimination among iNPH, AD, VD, PD, and PSP [69,70,71,72,73,74]. MRHI, AVV, and IPA are helpful in the discrimination between iNPH and PSP [46,76], as enlargement of the interpeduncular cistern due to a dilated third ventricle in iNPH and midbrain atrophy in PSP are both associated with reduced midbrain area, making classic MRI markers of midbrain and SCP atrophy unable to help towards that direction [75,76].

Regarding the association of imaging markers with the severity of symptoms, tap test, and shunt responsiveness, the following conclusions can be drawn. EI and CA have been, according to many studies, correlated to both tap test and shunt responsiveness [16,79,80,81,85]. Nevertheless, CA failed to predict shunt responsiveness according to Skalický et al., (2021) [83]. The load of white matter lesions has been negatively correlated to the tap test response [87], whereas a post-shunt decrease of periventricular white matter hyperintensities has been proposed as a potential marker for shunt responsiveness [90]. High tight convexity, Sylvian fissure dilation [85,86], wide temporal horns [16], focally dilated sulci [86], “empty sella” sign and “mismatch” sign [91], disproportionately enlarged subarachnoid spaces [16,93], and fewer lacunes [93] have also been correlated to shunt responsiveness.

Results of correlation of composite imaging scales such as Radscale [80,101,102,103,104] or DESH score [49,83,86,101] to either tap test or shunt responsiveness are rather inconclusive. Aqueductal CSF flow measurements using phase-contrast MRI, along with flow void sign may be helpful in predicting outcome after shunt [29,98,99], although ASV alone does not seem to predict shunt responsiveness [47]. Microstructural changes of white matter, as assessed using DTI, could be useful in the prediction of tap-test responder status, while specific patterns of these changes may correspond to specific symptom domains in iNPH patients [73,97]. Usage of brain volumetry based on 3D MRI images has proposed specific patterns of cortical thinning as a possible imaging marker for the prediction of tap test responsiveness [45]. Regarding functional imaging, postoperative improvement in glucose cortical metabolism using 18F FDG PET or normalization of dopaminergic deficit using DaT-Scan could be used as imaging markers for the assessment of shunt responsiveness [108,110]. Nevertheless, some studies concluded that preoperative imaging markers are either unable to predict outcome [90,100] or are inappropriate to be used as exclusion criteria [27] regarding shunt surgery.

We need to recognize the fact that there is a great heterogeneity among studies, regarding for instance the modality used, or in many cases, even the inclusion criteria used for the characterization of the patient cohorts. Another limitation of these studies, which should also be noted, is the fact that many of the markers are visually assessed, increasing the possibility of inter-rater and intra-rater variability.

## 5. Conclusions

As iNPH diagnosis is based on clinic-radiological features, neuroimaging is of paramount importance. There is a pleiad of signs, markers, indexes, and scores that are evaluated using different modalities. Some of them seem to be present in various neurological diseases, while others tend to be more disease-specific. Such imaging markers can also be useful in the differential diagnosis between iNPH and its mimics. Furthermore, some of these markers seem to correlate either with the severity of specific symptoms or with the tap test responsiveness status, while others seem to be able to predict the post-shunt clinical outcome. Given also the fact that they are non-invasive, such biomarkers could be used in the early and better selection of patients that could benefit from surgical treatment. Despite the above, and because of the relatively low negative prognostic value that most of these markers have, they should not be used to exclude patients from VL shunt placement. Future studies could possibly find better composite scales or even new imaging markers with better sensitivity and specificity, which would assist even more in the timely and differential diagnosis and management of this potentially reversible neurological syndrome.

## Figures and Tables

**Table 1:** Imaging markers that assist in the differential diagnosis of iNPH from its mimics.

iNPH Imaging Marker (s)	iNPH Mimic (s)	Reference (s)
CA	AD	[21]
Simplified CA	AD, DLB	[22]
ACA	AD	[23]
CA, simplified composite imaging scale consisting of EI, CA, focally enlarged sulci, and enlarged sylvian fissures	VD	[62]
Extent and pattern of hippocampal atrophy and peri-hippocampal dilation	AD	[24,64,65]
White matter lesion distribution	AD	[67]
Microstructural changes in white matter, evaluated using MRI DTI	AD, VD	[69]
	AD, PD	[70]
	AD, PSP	[71]
	PD	[72]
	AD	[73,74]
IPA	PSP	[76]
MRHI	PSP	[46]
Combination of EI and CA	PSP	[78]

iNPH: idiopathic normal pressure hydrocephalus; CA: callosal angle; ACA: anterior callosal angle; EI: Evans index; MRI: magnetic resonance imaging; DTI: diffusion tensor imaging; IPA: interpeduncular angle; MRHI: magnetic resonance hydrocephalic index; AD: Alzheimer’s disease; DLB: dementia with Lewy bodies; VD: vascular dementia; PSP: progressive supranuclear palsy.

**Table 2:** Preoperative imaging markers of iNPH and their potential prognostic value.

Preoperative iNPH Imaging Marker (s)/Index/Score	Potential Prognostic Value	Reference (s)
EI higher than 0.3	Better shunt surgery outcome	[16,21]
More acute CA	Better shunt surgery outcome	[16,21,79]
More acute CA	Positive tap test responder status	[80]
ACA	Better shunt surgery outcome	[84]
High tight convexity	Clinical improvement one year after shunt	[85]
Wide temporal horns	Better shunt surgery outcome	[16]
High tight convexity, dilated Sylvian fissures, and focally dilated sulci	Subjective shunt response at 1-month follow-up	[86]
White matter lesions load	Negative correlation with tap test responsiveness	[87]
Plentiful PVH and DWMH	Improvement in gait and cognitive performance after shunt placement	[68]
“Empty sella” sign and “mismatch” sign	Positive tap test responder status	[91]
DESH sign	Better shunt surgery outcome	[16,93]
Fewer lacunes	Better shunt surgery outcome	[93]
Decrease in ADC	Better shunt surgery outcome	[94]
Signs of subdural hemorrhage	Worse shunt surgery outcome	[94]
Microstructural changes of white matter using MRI DTI	Tap test responder status	[42,73]
Measurement of aqueductal flow	Shunt surgery outcome	[29,98,99]
DESH score	Better shunt surgery outcome	[49]
DESH score	Subjective shunt response at 1-month follow-up	[86]
Radscale	Shunt surgery outcome	[102]
Radscale total-score (higher)	Better shunt surgery outcome	[103]
Patterns of cortical thickness in MRI based volumetry	Tap test responder status	[45]
Dopaminergic deficit in DaTScan	iNPH pathology progression	[110]

iNPH: idiopathic normal pressure hydrocephalus; CA: callosal angle; ACA: anterior callosal angle; EI: Evans index; DESH: disproportionally enlarged subarachnoid spaces hydrocephalus; MRI: magnetic resonance imaging; DTI: diffusion tensor imaging.

## Data Availability

Not applicable.

## Abbreviations

3D: 3-dimentional; ACA: anterior callosal angle; AC-PC: anterior commissure—posterior commissure; AD: Alzheimer’s disease; ADC: apparent diffusion coefficient; ALVI: anteroposterior diameter of the lateral ventricle index; ASV: aqueductal stroke volume; ATR: anterior thalamic radiation; AVV: automated ventricular volumetry; BD: Binswanger disease; BVR: brain-to-ventricle ratio; CA: callosal angle; CBD: corticobasal degeneration; CBF: cerebral blood flow; CCD: callosal-commissural distance; CgH: cingulum-hippocampus; CH: callosal height; CMW: cella media of the lateral ventricle width; CSF: cerebrospinal fluid; CT: computed tomography; CTR: cella media-to-temporal horn ratio; CTW: collateral trigones width; CVD: callosal-ventricular distance; D a: axial diffusivity; D r: radial diffusivity; DaT-Scan: dopamine transporter single photon emission; DESH: Disproportionately enlarged subarachnoid space hydrocephalus; DLB: dementia with Lewy bodies; DMI: diffusion microstructure imaging; DTI: Diffusion Tensor Imaging; DWMH: deep white matter hyperintensity; DWML-V: deep white matter lesions volume; EI: Evans index; FA: fractional anisotropy; FHD: frontal horn diameter; FOR: frontal and occipital horn ratio; FTD: frontotemporal dementia; FVP: flow void phenomenon; IFO: inferior fronto-occipital fasciculus; ILF: inferior longitudinal fasciculus; iNPH: idiopathic Normal pressure hydrocephalus; IPA: interpeduncular angle; ISD: inner skull diameter; LP: lumbo-peritoneal; mCMI: modified cella media index; MD: mean diffusivity; MRE: magnetic resonance elastography; MRHI: magnetic resonance hydrocephalic index; MRI: magnetic resonance imaging; mRS: modified Rankin Scale; MSA-P: multiple system atrophy parkinsonian type; MSID: maximum supratentorial intracranial diameter; PD: Parkinson’s disease; PSP: progressive supranuclear palsy; PWML-V: periventricular white matter lesions volume; rCBF: regional cerebral blood flow; SCP: superior cerebellar peduncle; SVW: vertical width of the supraventricular brain; THW: temporal horns width; VD: vascular dementia; VL: ventrolateral; VPL: ventroposterolateral; VRSs: Virchow-Robin spaces; WML: white matter lesions.
